# Proteolysis-targeting chimeras (PROTACs) in cancer therapy

**DOI:** 10.1186/s12943-021-01434-3

**Published:** 2022-04-11

**Authors:** Xinyi Li, Wenchen Pu, Qingquan Zheng, Min Ai, Song Chen, Yong Peng

**Affiliations:** 1 Laboratory of Molecular Oncology, Frontiers Science Center for Disease-related Molecular Network, National Clinical Research Center for Geriatrics, West China Hospital, Sichuan University, Chengdu, 610064 China; 2 State Key Laboratory of Oral Diseases, National Clinical Research Center for Oral Diseases, Department of Orthodontics, West China Hospital of Stomatology, Sichuan University, Chengdu, 610064 China

**Keywords:** PROTAC, Targeted cancer therapy, Ubiquitin-proteasome system, Protein degradation

## Abstract

Proteolysis-targeting chimeras (PROTACs) are engineered techniques for targeted protein degradation. A bifunctional PROTAC molecule with two covalently-linked ligands recruits target protein and E3 ubiquitin ligase together to trigger proteasomal degradation of target protein by the ubiquitin-proteasome system. PROTAC has emerged as a promising approach for targeted therapy in various diseases, particularly in cancers. In this review, we introduce the principle and development of PROTAC technology, as well as the advantages of PROTACs over traditional anti-cancer therapies. Moreover, we summarize the application of PROTACs in targeting critical oncoproteins, provide the guidelines for the molecular design of PROTACs and discuss the challenges in the targeted degradation by PROTACs.

## Introduction

Targeted cancer therapies aim to target cancer-associated biomolecules (such as oncoproteins) and interfere with their oncogenic cellular processes in cancer tissues. In the past several decades, targeted therapies have achieved remarkable advances in cancers and become a powerful treatment strategy for cancer patients. For example, small molecular inhibitors or monoclonal antibodies have been successfully developed to target overexpressed or overactivated proteins in cancer [1]. However, due to limited therapeutic benefit, drug resistance and off-target effect of these targeted therapies, researchers are still seeking more effective and specific strategy to target cancer-related oncoproteins.

Inspired by the fact that cells employ the ubiquitin-proteasome system (UPS) to maintain intracellular protein homeostasis, Deshaies laboratory designed and synthesized the functional molecule Protac-1 to induce the degradation of methionine aminopeptidase-2 (MetAP-2) via recruiting UPS in 2001. Protac-1 consists of three covalently-linked segments: a domain containing the IκBα phosphopeptide that is recognized by Skp1-Cullin-F-box complex (SCF, an E3 ligase to initiate protein ubiquitination and degradation by UPS), a domain having ovalicin (MetAP-2 inhibitor), and a linker connecting these two domains [2]. This work proposed the initial concept of proteolysis-targeting chimeras (PROTACs), an engineered technique that induces degradation of protein of interest (POI) via UPS in living cells. Subsequently, researchers developed different peptide-based PROTACs to eliminate the disease-promoting proteins, such as androgen receptor (AR), estrogen receptor (ER), FK506 binding protein (FKBP12) and aryl hydrocarbon receptor (AHR) [3–6]. Because the peptide backbones have low lipophilicity (unfavorable to cross cell membrane) and are easily hydrolyzed by digestive enzymes, these peptide-based PROTACs have poor cell permeability and low stability, limiting their application.

Given that some small chemical molecules exhibit stronger lipophilicity, Crews’ group developed the first small-molecule based PROTAC in 2008 to effectively degrade AR in cancer cells. This cell permeable PROTAC comprises the chemical nutlin (the E3 ligase MDM2 inhibitor) and a non-steroidal AR ligand (SARM), connected by a PEG-based linker [7]. In 2010, Itoh et al. utilized the chemical methyl bestatin to synthesize another PROTAC molecule, thus recruiting the E3 ligase inhibitor-of-apoptosis-protein (IAP) to degrade POI [8]. To increase potency and target selectivity, small molecules with high affinity and specificity, such as phthalimides recruiting the E3 ligase cereblon (CRBN) [9–14] or VHL-1 recognizing the E3 ligase Von Hippel-Lindau (VHL) [15–17], were introduced into PROTACs to downregulate numerous cancer targets, such as Ikaros family zinc finger protein 1/3 (IKZF1/3) and estrogen-related receptor alpha (ERRα). The breakthroughs of small molecule-based PROTACs pave the way for PROTACs as therapeutic anticancer strategies.

Recently, a series of novel PROTACs have been developed to expand their applications with more advantages, such as RNA-PROTAC for degrading undruggable RNA-binding proteins [18], PhotoPROTAC for optical control of protein degradation [19–25], and CLIPTAC for increasing bioavailability [26]. Importantly, PROTAC is a highly promising technology for clinical applications, given that Arvinas Therapeutics Company has initiated the first-in-human trial in 2019 (i.e., PROTAC ARV-110 targeting AR for the treatment of prostate cancer), and at least 15 targeted degraders are expected to enter clinical trials by the end of 2021 [27].

In this review, we introduce the principle and development of PROTAC technology and summarize the application of PROTACs in targeting crucial oncoproteins. Furthermore, we discuss the challenges in PROTAC realm and propose the guidelines to design excellent PROTACs for targeted cancer therapy.

## Principle of PROTACs

### PROTACs hijack the ubiquitin-proteasome system (UPS)

**Fig. 1:**
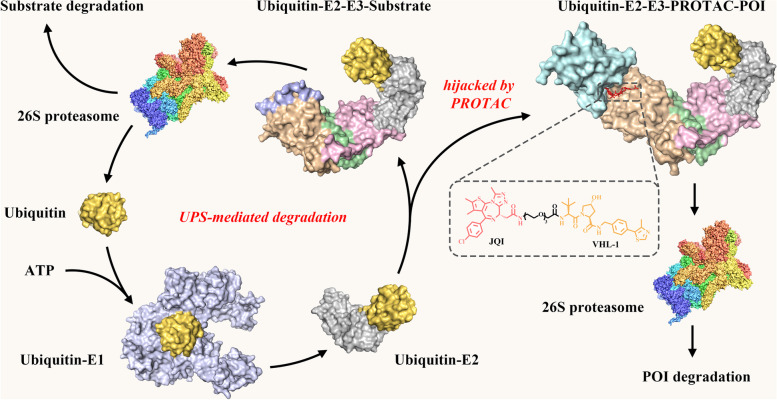
The mechanism of PROTACs based on the UPS. UPS consists of specific enzymes (E1, E2 and E3) modifying proteins with ubiquitin and the proteasome degrading the ubiquitin-tagging proteins. PROTAC contains a POI ligand, an E3 ligand and a linker. The E3-PROTAC-POI ternary complex induces the polyubiquitination and proteasome-mediated degradation of POIs. The presented PROTAC is BRD4 degrader MZ1 that is composed of POI ligand JQ1 (red) and E3 ligand VHL-1 (yellow)

UPS is a highly conserved mechanism for degradation of both normal and misfolded proteins in eukaryotic cells, thus keeping intracellular protein homeostasis [28–30]. In UPS, proteins to be degraded are covalently tagged with ubiquitin (Ub, a 76-amino acid protein), and this tagging process is catalyzed by three enzymes known as Ub-activating enzyme (E1), Ub-conjugating enzyme (E2) and Ub-ligase (E3): free Ub is activated by E1 and then attached to the cysteine residue (Cys) of E1 to form a thioester bond via an ATP-dependent reaction; the Ub-tagged E1 transfers its Ub to the Cys of E2 through a trans-thioesterification reaction; E3 recruits Ub-tagged E2 and E3 substrate to label the ubiquitin at the lysine residue (Lys) of the substrate. Such repeated ubiquitination processes generate a poly-Ub chain (mainly linked through Lys48 of Ub) on the target protein, which guides the substrate to 26S proteasome for degradation [31, 32] (Fig. 1). In human proteome, there are two E1s, about forty E2s and more than 600 E3s. Among them, the E3 ligases are responsible for specifically recognizing substrates.

Inspired by UPS, researchers designed PROTACs to hijack the UPS and degrade POI. PROTAC molecule consists of three covalently-bonded moieties: a ligand to bind POI (POI ligand), another ligand to recognize E3 ligase (E3 ligand) and a linker to conjugate the two ligands. PROTAC simultaneously recruits E3 ligase and POI, forming the “E3-PROTAC-POI” ternary complex. Gadd et al. solved the crystal structure of bromodomain-containing protein 4 (BRD4) PROTAC MZ1 in complex with human VHL and BRD4 bromodomain, supporting the formation of the ternary complex [33]. This complex potentiates the substrate recognition by E3 ligase and promotes the transfer of Ub to POI, accelerating the poly-ubiquitination and subsequent proteasome-mediated degradation of POI [34] (Fig. 1).

### Hook effect of PROTAC

The bifunctional molecule (“B”) interacts with its two substrates (“A” and “C”), forming “A-B-C” ternary complex to exert its biological functions. When the concentration of “B” exceeds a certain range, “B” prefers to form “A-B” and “B-C” binary complexes, instead of “A-B-C” ternary complex, thus reducing the activity of “B”. This phenomenon is termed as the “hook effect” [35]. As a bifunctional molecule, high-dose of PROTAC tends to form “PROTAC-POI” and/or “PROTAC-E3” complexes rather than “POI-PROTAC-E3” ternary complex (required for POI degradation), thus reducing its degradation potency [36–39]. Hook effect exists in most known PROTACs, thereby this effect is available to check whether the synthesized PROTAC is bifunctional.

To avoid hook effect, a wide range of PROTAC concentrations should be tested in cellular activity assays to determine the maximal concentration without hook effect [40]. Unfortunately, the research on hook effect of PROTACs *in vivo* is lacking, so it’s hard to choose an appropriate concentration of PROTAC in *in vivo* application [41, 42]. Intriguingly, some PROTACs could trigger the positive cooperative assembly of ternary complexes by inducing “neocontacts” between E3 and POI (e.g., “neocontacts” between VHL and BRD4 caused by PROTAC MZ1). These “neocontacts” stabilize “POI-PROTAC-E3” ternary complex and increase the threshold for triggering the hook effect [33]. Therefore, optimizing the PROTAC structure to enhance this “neocontacts” is a potential method to avoid hook effect to some extent.

### Advantages and disadvantages of PROTAC

Diverse therapeutic strategies, such as small-molecule inhibitor, monoclonal antibody, RNA interference and CRISPR/Cas9, have been developed to treat human cancers [43–46]. The unique chemical and biological features of PROTAC endow it with advantages and disadvantages in cancer therapy (Table 1).

**Table 1:** Comparisons of different targeted cancer therapies

	PROTAC	CRISPR/Cas9	RNA interfering	small-molecule inhibitor	monoclonal antibody
Requirement of active sites	No	No	No	Yes	Yes
Elimination of pathogenic proteins	Yes	Yes	Yes	No	No
Undruggable targets	Yes	Yes	Yes	No	Yes
Tissue penetration	Moderate	Poor	Poor	Yes	Poor
Intracellular targets	Yes	Yes	Yes	Yes	No
Systemic delivery	Yes	Poor	No	Yes	Yes
Catalytic mechanism of action	Yes	Yes	Yes	No	No
Route of administration	PO/IV/SC	IV	IV/SC	PO/IV/SC	IV/SC

Note: *IV* intravenous injection, *PO* peros, *SC* Subcutaneous injection

#### Advantages of PROTAC

##### Event-driven mechanism

The activity of small molecule drugs, especially the FDA-approved inhibitors, is usually driven by occupancy of target (called “occupancy-driven mechanism”), while PROTACs act as catalysts to initiate degradation event of target protein in a repeatable manner (called “event-driven mechanism”) [36]. Thus, one equivalent of PROTAC could degrade multiple equivalents of POI, allowing the dosage, administration frequency and toxicity of PROTACs lower than those of small-molecule drugs. Additionally, due to the catalytic behavior of PROTAC, transient or low-abundance ternary complexes are sufficient to achieve target degradation. Therefore, ligands with lower POI/E3 affinity and high selectivity are favorable for PROTAC activity, enabling the rapid assembly/disassembly of functional ternary complex. Moreover, PROTACs eliminate the whole functions of targets, overcoming the therapeutic challenges (commonly occurred in the treatment by small-molecule inhibitors) caused by the non-catalytic functions or gain/loss-of-function mutations of POIs [47].

##### Degrading “undruggable” targets

Many proteins, such as DNA-binding proteins (DBPs, e.g. transcriptional factor c-myc) and RNA-binding proteins (RBPs, e.g. IGF2BPs), play important roles in cancer initiation and progression and are regarded as high-value therapeutic targets. But these proteins generally lack targetable pockets (orthosteric or allosteric sites), so they are deemed “undruggable” by small-molecule inhibitors. PROTACs could use the low-affinity small-molecule ligands (transiently associated with the possible binding site of POI) or the oligonucleotides as protein decoys to release the dependence on the well-defined targetable pockets, providing opportunities to degrade “undruggable” proteins [48].

##### Avoiding compensatory protein expression

Targeted therapies, such as small-molecule inhibitors, may trigger compensatory protein expression after administration, which decreases drug efficacy and increases side effects [49]. For instance, treatment with statin, HMG-CoA reductase (HMGCR) inhibitor, increased HMGCR level by enhancing gene transcription and retarding protein degradation, thus attenuating statin’s activity to treat cardiovascular diseases [50]. PROTAC can potently downregulate POI protein level through accelerating UPS-mediated degradation, thus offering a pathway to prevent compensatory protein expression of POI. Moreover, genetical interference with short hairpin RNA might induce a secondary cellular response (e.g., by triggering the compensatory mechanism) to maintain cell homeostasis, so it is difficult to disclose the *bona fide* function of proteins. Because of acute and reversible depletion of protein, PROTAC could be a molecular tool to dissect protein function [40].

#### Disadvantages of PROTAC

PROTAC needs to enter cells to mobilize intracellular UPS, so its membrane permeability is the key to PROTAC’s function. Currently, the penetration mechanism of PROTAC has not yet been elucidated. Most known PROTACs have the molecular weights (M.W.) of 1000–2000 Da [51, 52], so they penetrate cell membrane mainly through passive diffusion and active transport. Nevertheless, large M.W. and large exposed polar surface area of PROTACs makes their cell/tissue permeability worse than small molecules. Various strategies have been employed to improve permeability of PROTACs. The common ways are to limit its M.W. below 1000 Da [53] or to split the molecule into two smaller precursors and generate mature PROTAC in cells (CLIPTAC) [26]. Additionally, the cell permeability of PROTAC could be increased by introducing long flexible linkers to form intramolecular hydrogen bonds that partially reduce polarity [54], or attaching cell-permeable peptides (such as poly-D-arginine sequence) to E3 ligands [3]. Except for modifying PROTAC itself, application of nanoparticles such as liposomes to deliver PROTAC also significantly enhanced the cellular uptake of PROTACs [55].

Currently, the design of PROTACs needs known POI/E3 ligands as protein decoys, so PROTAC development largely depends on the discovery and optimization of these ligands. Moreover, some known POI/E3 ligands exhibit low specificity, making such PROTACs have off-target effects [34]. Therefore, identifying highly specific POI/E3 ligands is critical for developing good PROTACs.

## Development of PROTAC technology

### Classification of PROTAC

According to the chemical structure of POI ligands, PROTACs could be divided into peptide-based, small molecule-based and nucleotide-based ones. Peptide-based PROTACs contain peptidic POI ligands mimicking the sequences of native POI-binding proteins. For example, Signal transducer and activator of transcription 3 (STAT3) is a critical transcription factor and its hyperactivation is tightly associated with cancer initiation and progression [56]. SI-109, a peptide stemmed from the STAT3-binding motif of the protein gp130, was utilized to develop a peptide-based PROTAC termed as SD-36, which achieved potent STAT3 degradation and inhibited leukemia and lymphoma *in vitro* and *in vivo* [57]. Peptide-based PROTACs have advantages in binding affinity, target specificity and chemical synthesis, while they suffer from limited membrane permeability and digestive intolerance [58]. Thus, peptide-based PROTAC is usually intravenously injected and especially suitable for the membrane proteins or the treatment of hematological diseases.

Small molecules, especially FDA-approved anticancer inhibitors, could be used as the POI ligands to build small molecule-based PROTACs. For instance, BRAF^V600E^ (a mutant of RAF kinase) prevalently occurs in melanoma and colorectal cancer, driving oncogenic ERK signaling even in the absence of activated RAS [59]. Posternak et al. introduced the small-molecule BRAF^V600E^ inhibitor BI882370 as POI ligand and pomalidomide as the E3 ligand for CRBN. The obtained PROTAC P4B exhibited effective BRAF degradation to inhibit melanoma and colon cancer harboring BRAF mutation [60]. Compared with peptide-based PROTAC, small molecule-based one displays improved cell permeability and resistance to digestion, thus allowing more manners of administration and expanded target scope. Notably, small-molecule ligands usually have poor target specificity [58], so more concerns should be given to this point when selecting or optimizing small-molecule POI ligands.

Recently, nucleotide-based PROTACs have been developed, which use oligonucleotides as POI ligands. Numerous RBPs (e.g. Lin28, IGF2BPs and Musashi-1/2) and DBPs (e.g. NF-κB, c-myc and STAT3) are overexpressed and/or overactivated in human cancers, promoting tumorigenesis and cancer development. But discovery of RBP/DPB-targeted drugs is challenging due to the lack of targetable binding pockets within RBPs/DBPs. For instance, Lin28 is a highly conserved RBP that promotes tumorigenesis by interacting with let-7 precursor (pre-let-7) to inhibit the biogenesis of let-7, a tumor suppressive microRNA [61]. But Lin28 doesn’t have well-defined targetable pockets for small-molecule intervention. Ghidini et al. utilized the Lin28-binding oligoribonucleotides derived from pre-let-7 as POI ligand to synthesize a nucleotide-based PROTAC termed RNA-PROTAC. This PROTAC accomplished remarkable Lin28 degradation in leukemia cells with high selectivity and negligible toxicity [18]. In addition, Samarasinghe et al. developed DNA-based PROTAC (termed TRAFTAC), which used DNA sequence as POI ligand to recognize the transcription factor NF-κB for targeted degradation [62]. These nucleotide-based PROTACs expand the concept of PROTACs and provide a novel strategy for cancer treatment.

### New concepts of PROTAC technologies

#### PhotoPROTAC

**Fig. 2:**
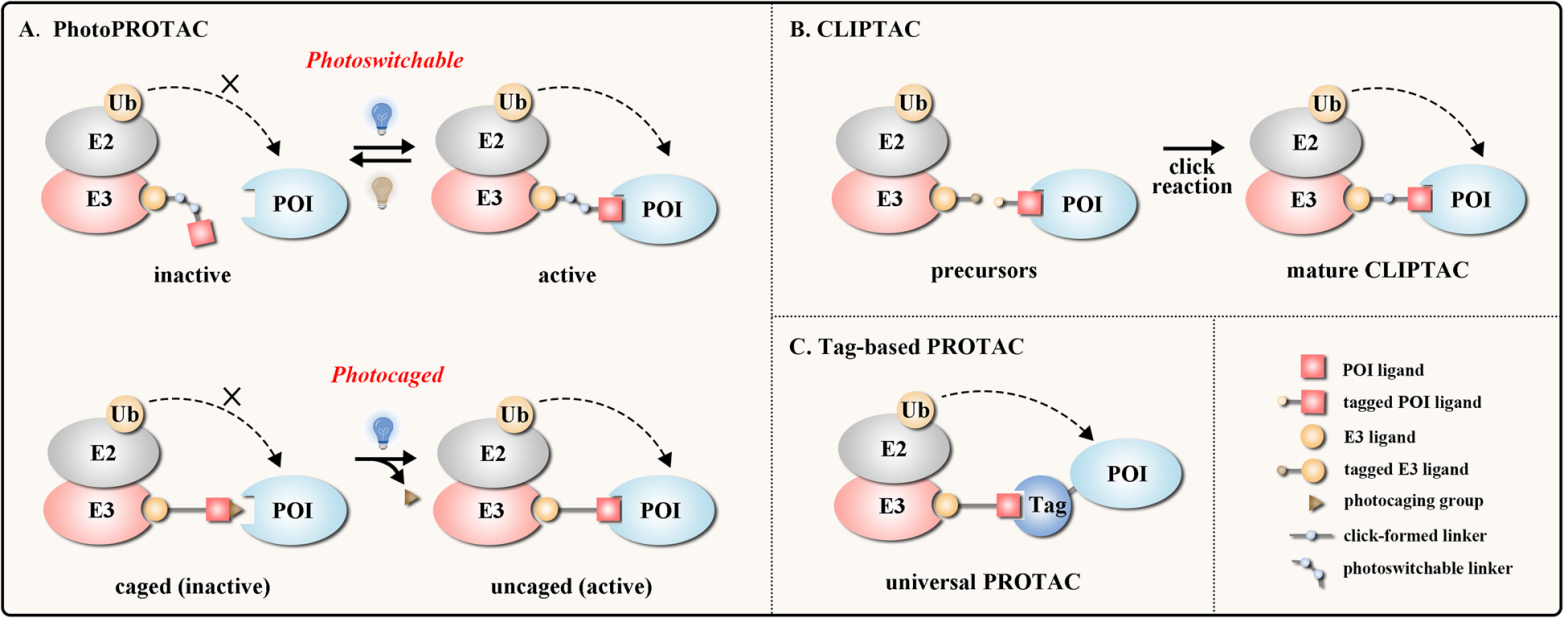
The emerging new concepts of PROTAC technologies. **a** photoswitchable PROTAC (upper) achieves reversible optical control of protein degradation by interconverting between inactive and active conformers, and photocaged PROTAC (lower) irreversibly achieves light-induced protein degradation by removing photocaging group. **b** CLIPTAC can be formed intracellularly through click combination of two tagged precursors. **c** HaloPROTAC and dTAG system utilize tag fusion POI and ligand that bind to tag protein

Through optically-controlled generation or release of active small-molecule modulators, light with high spatiotemporal resolution has been widely used in biomedical research and disease treatment [63]. Some moieties (e.g. azobenzene) within molecules could be reversibly or irreversibly changed under light stimulation, altering the spatial configuration and the physical/chemical/biological properties of molecules. This concept inspired the development of PhotoPROTAC, which utilized the photoswitches [64] or the photocages [65] to realize the spatiotemporal control of PROTAC function (Fig. 2a).

Photoswitchable PROTACs optically control protein degradation in a reversible manner by using a photoswitchable moiety (e.g. azobenzene) on linker or E3 ligand. Without light irradiation, PROTAC maintains the inactive conformation that is unable to form a stable ternary complex. Upon light exposure at the designed wavelength, PROTAC switches to the active conformation, forming a functional ternary complex to degrade target [19–21]. For example, Reynders et al. designed PHOTACs involving azobenzene moiety on the linker to degrade BET family proteins and suppress acute lymphoblastic leukemia (ALL) cells in the presence of 390 nm UV light [21]. Notably, with appropriate light exposure (e.g. 500 nm for azobenzene-containing PROTAC), target degradation could be halted by converting the PROTAC into an inactive conformation [19–21].

Photocaged PROTACs irreversibly accomplish protein degradation by incorporating photolabile blocking groups (e.g. nitroveratryloxycarbonyl group, NVOC). Without light stimulation, the photocaging group labeling to the E3 ligand impairs the binding between PROTAC and E3 ligase. Upon light exposure, the photocaging group is released from PROTAC, benefiting the formation of POI-PROTAC-E3 ternary complex [22–25]. For instance, Liu et al. used NVOC on CRBN ligand to synthesize photocaged PROTACs. These obtained opto-PROTACs were able to degrade IKZF1/3, BRDs or ALK fusion protein (using corresponding POI ligands) upon 365 nm UV irradiation, inhibiting cancer cell proliferation in an optical-controlled manner [22].

#### CLIPTAC

PROTACs usually have large M.W., limiting their solubility, pharmacokinetics and bioavailability, thus how to reduce the M.W. of PROTACs is critical. Click chemistry, coined by Sharpless group, is used to describe chemical reactions with the advantages of benign reaction condition, high yielding, high selectivity as well as wide scope [66]. To date, click chemistry has developed as a fundamental technology to covalently modify biomolecules under physiological conditions, which is particularly suitable for building conjugated skeletons from two small precursors in cells [67, 68]. Inspired by the concept of click chemistry, Lebraud et al. prepared a tetrazine-tagged E3 ligand and a *trans*-cyclooctene-tagged POI ligand as the precursors. Via the click reaction between tetrazine and *trans*-cyclooctene, generating a covalent six-membered ring moiety, these two precursors formed integrated PROTACs (termed as CLIPTAC) in cells (Fig. 2b) that successfully degraded oncogenic BRD4 or ERK1/2 [26]. Therefore, the CLIPTAC has become an attractive solution for reducing the M.W. of PROTACs.

#### Tag-based PROTAC

The *ab initio* development of PROTAC is a time-consuming and multistep process, including molecular design, chemical synthesis and cell−/animal-based evaluation [51]. The selection of appropriate E3 ligase/E3 ligand system is crucial for the progress of PROTAC research. However, there are more than 600 E3 ligases in human proteome and the atlas for POI-E3 ligase interactions is far from clear. Thus, researchers have established the tag-based PROTAC systems, in which the tag-POI fusion protein was expressed in cells and the universal PROTAC molecule was administrated to recruit the candidate E3 ligase and the tag of tag-POI protein. Measuring the abundance of tag-POI protein was able to verify whether the candidate E3 ligase could initiate POI degradation. The most widely-used tag-based PROTACs are HaloPROTAC and dTAG (Fig. 2c) [69–72]. These tag-based PROTACs suggest promising molecular tools to check whether a candidate E3 ligase/E3 ligand system is suitable for PROTAC, but they could not be used as the therapeutics for disease treatment.

## PROTACs in targeted cancer therapy

Cancer initiation and progression is a complex process characterized by sustaining proliferative signaling, evading growth suppressors, resisting cell death, inducing angiogenesis, activating invasion and metastasis [73]. Compelling evidence has demonstrated that some overexpressed and/or overactivated proteins play crucial roles in tumorigenesis and act as potential therapeutic targets. Here we summarize the applications of PROTACs in targeted cancer therapy.

### Targeting cancer cell proliferation

**Fig. 3:**
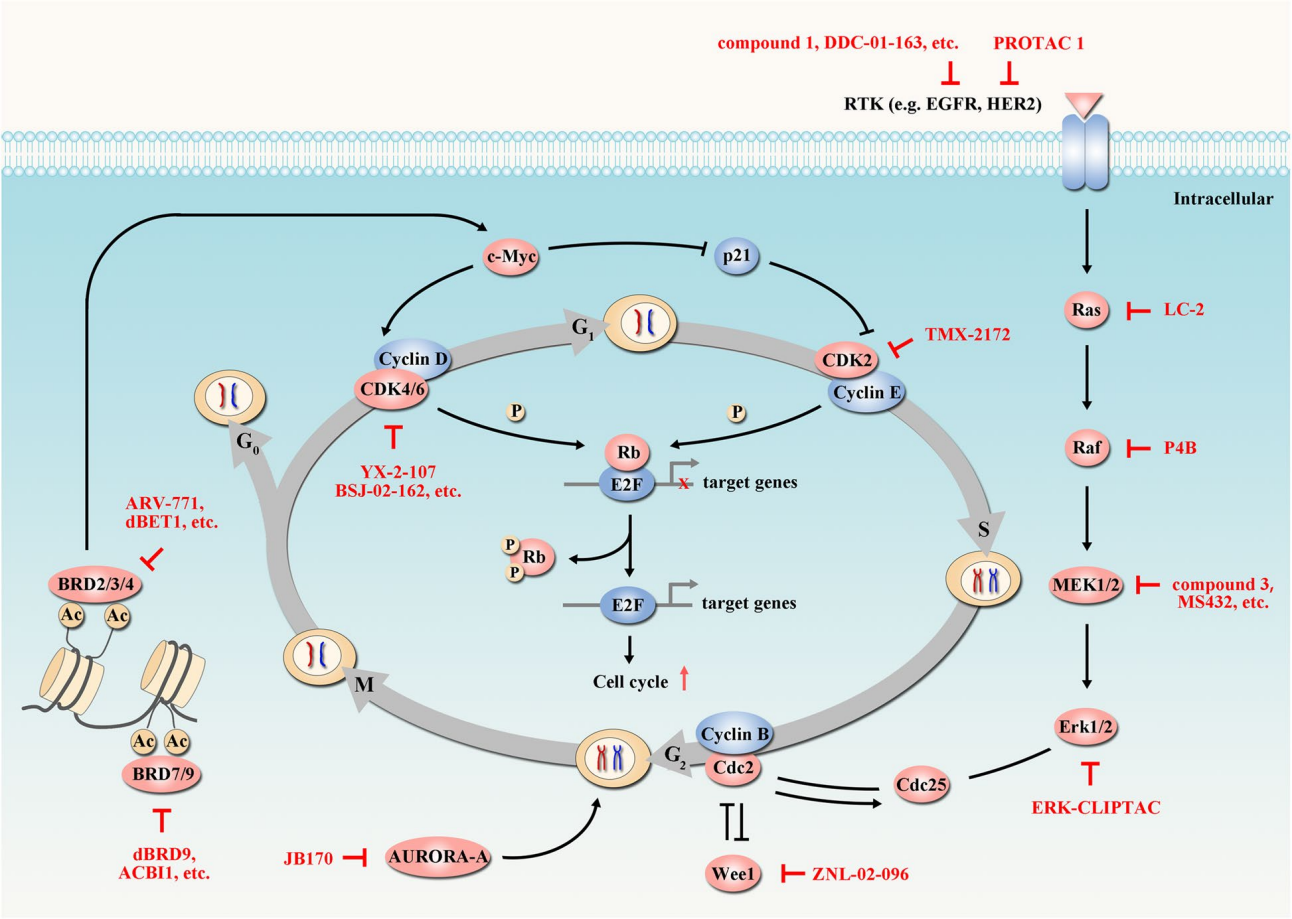
PROTACs targeting cancer proliferation. In cell cycle regulation, some proteins (e.g. c-Myc, p21) act as accelerator or inhibitor to regulate CDK expression. CDKs and their chaperones phosphorylate retinoblastoma protein (Rb), thus releasing transcription factor E2F and promoting DNA replication. The RAS-RAF-MEK-ERK pathway also plays a central role in growth-promoting signaling and elicits cell cycle progression. These key elements in cancer proliferation can be targeted by diverse PROTACs (red arrow). Tumor-suppressor proteins are indicated in blue and oncogenic proteins are indicated in red. In the presented pathways, PROTACs have been developed targeting BRD4 [12, 76], CDK4/6 [77, 78], EGFR [79, 80], AURORA-A [81], Raf [60], BRD7/9 [82, 83], CDK2/5 [84], ERK1/2 [26], HER2 [79], MEK1/2 [85, 86], Ras [87] and Wee1 [88]

The growth-promoting signals, including RAS-RAF-MEK-ERK pathway, are frequently hyperactivated in tumors, eliciting cell cycle progression to induce the uncontrolled cell proliferation [74, 75] (Fig. 3). PROTAC technology has been applied to target the overexpressed, overactivated or mutated proteins involved in cell cycle regulation (Table 2).

**Table 2:** The structures of PROTAC molecules targeting cell proliferation in cancers. (red: POI ligand; yellow: E3 ligand)

Target	PROTAC	PROTAC structure	Cancer	Ref	Target	PROTAC	PROTAC structure	Cancer	Ref
BRD4	ARV-825		BL	[39]	BRD4	SNIPER(BRD4)-1		BC	[89]
BRD4	dBET1		AML	[12]	BRD4	A1874		CRC, M, BL, et al.	[90]
BRD4	MZ1		CC	[33, 91]	BRD4	CLIPTAC-BRD4		CC, M, CRC	[26]
BRD4	dBET6		T-ALL	[92]	BRD4	opto-dBE T1		MM, PC, ALCL, et al.	[22]
BRD4	ZXH-3-26		MM	[93]	BRD4	PROTAC4		BC, PC	[23]
BRD4	ARV-771		PC	[76]	BRD4	pc-PROTAC1		BL, HCC	[25]
BRD4	macroPROTAC-1		PC, AML	[94]	BRD4	PHOTAC-I-3		ALL	[21]
BRD4	BETd-246		TNBC	[95]	BRD4	photoPROTAC-1		BL	[20]
BRD4	BETd-260		AML, ALL	[96]	BRD4	PROTAC3		CC	[24]
BRD4	KB02-JQ1		/	[97]	EGFR	compound 1		OC, CC	[79]
BRD4	XH2		BC	[98]	EGFR	compound 14o		NSCLC	[99]
BRD4	DP1		DLBCL	[100]	EGFR	compound P3		LC	[101]
CDK4/6	BSJ-03-123		AML	[102]	EGFR	MS154		LC	[103]
CDK4/6	pal-pom		TNBC	[104]	EGFR	DDC-01-163		LC	[80]
CDK4/6	BSJ-02-162		MCL	[77]	AURORA-A	JB170		AML, OS, NB, HCC	[81]
CDK4/6	BSJ-04-132		T-ALL	[77]	Raf	P4B		M, CRC	[60]
CDK4/6	YX-2-107		ALL	[78]	AR	PROTAC 14		CC	[7]
CDK4/6	compound 11		M	[105]	AR	ARCC-4		PC	[106]
CDK4/6	PROTAC 34		ALL, AML, MM, TNBC	[107]	AR	TD-802		PC	[108]
AR	MTX-23		PC	[109]	BRD7/9	ACBI1		AML, M, NSCLC	[83]
AR	ARD-69		PC	[110]	CDK2/5	TMX-2172		OC	[84]
AR	SNIPER(AR)-51		PC	[111]	CDK8	JH-XI-10-02		T-ALL	[112]
ALK	MS4077		NSCLC, ALCL	[113]	CDK9	PROTAC 3		CRC	[114]
ALK	TL13–12		ALCL, NB, NSCLC	[52]	CDK9	THAL-SNS-032		T-ALL	[38]
ALK	opto-dALK		NSCLC, ALCL	[22]	CDK9	B03		AML	[115]
ALK	SIAIS117		NSCLC, ALCL	[116]	CDK9	compound F3		PC	[117]
BLK	PROTAC 7		CML	[118]	Cdc20	CP5V		BC	[119]
BRD7/9	dBRD9		AML	[82]	c-Met	PROTAC 7		TNBC, GC	[79]
BRD7/9	VZ185		MRT	[120]	CREPT	PRTC		PaC	[121]
CYP1B1	compound 6C		PC	[122]	MEK1/2	MS432		CRC, M	[86]
DHODH	probe 10		PaC	[123]	Ras	LC-2		NSCLC, PaC	[87]
ER	ERD-308		BC	[124]	GSPT1	CC-885		AML	[125]
ER	compound I-6		BC	[126]	PLK1	HBL-4		AML	[127]
ER	TD-PROTAC		BC	[128]	SLC9A1	d9A-2		CML	[129]
ER	SNIPER(ER)-87		BC	[89]	TACC3	SNIPER(TACC3)-1		FS, OS, CRC	[130]
ERK1/2	ERK-CLIPTAC		CC, M, CRC	[26]	TRIM 24	dTRIM24		AML	[131]
FLT-3	FLT-3 PROTAC		AML	[132]	TRKA/C	CG416		CRC, AML	[133]
HER2	PROTAC 1		OC, BC	[79]	Wee1	ZNL-02-096		OC, T-ALL	[88]
MEK1/2	compound 3		M	[85]	α1A-AR	α1A-AR		PC	[134]

#### BRD4

BRD4, a member of the bromodomains and extraterminal (BET) family, is an epigenetic reader of histone acetylation, triggering the transcription of pro-proliferative genes, such as c-myc [135]. Small-molecule BRD4 inhibitors including JQ1 and BETi-211 can downregulate c-myc level and induce potent anti-proliferative response [136]. However, high dose of BRD4 inhibitors is required to ensure sufficient BRD4 inhibition [39] and their antitumor efficacy might be unsatisfactory only by disrupting the bromodomain of BRD4 [12].

In 2015, Bradner’s group used JQ1 and thalidomide (a ligand for CRBN E3 ligase) to develop the BRD4-targeting PROTAC dBET1 with a DC_50_ value of 430 nM, attenuating tumor progression *in vitro* and *in vivo* by reducing the expression of BRD4 and c-myc [12]. To improve the degradation potency, Hines et al. synthesized the nutlin-base PROTAC A1874 to recruit MDM2 E3 ligase to effectively degrade BRD4 with DC_50_ of 32 nM [90]. Moreover, by utilizing VHL ligand and replacing the “(CH_2_CH_2_O)_3_” moiety of A1874 linker with “CH_2_CH_2_OCH_2_CH_2_CH_2_O”, the new PROTAC ARV-771 exhibited rapid BRD4 degradation (DC_50_ value < 1 nM) and potent antitumor effects in castration-resistant prostate cancer [76]. JQ1’s optimized analogue OTX015 was also applied to PROTAC ARV-825 to obtain a DC_50_ value of < 1 nM, leading to prolonged c-myc loss and enhanced anti-proliferative effects in Burkitt’s lymphoma cells [39].

As JQ1 and OTX015 were non-selective BRD4 inhibitors, dBET1 and ARV-825 also caused the degradation of both BRD2 and BRD3. Intriguingly, Zengerle et al. described a JQ1-based PROTAC MZ1, choosing VH032 as VHL ligand, exhibiting preferential degradation of BRD4 over BRD2/3 in cervical cancer cells [91]. This evidence indicated that PROTAC might gain selectivity, even starting with non-selective ligands. Recently, Gadd et al. resolved the crystal structure of BRD4-MZ1-VHL ternary complex, which suggested a BRD4-VHL “neocontacts” resulted from the MZ1-induced cooperative recognition [33]. Nowak et al. demonstrated that such “neocontacts” were plastic and generated several distinct BRD4-VHL conformations. Suitable length of PROTAC linker could reinforce the cooperative interaction between BRD4 and VHL, thereby conferring PROTAC the selectivity toward BRD4. This finding guided the development of BRD4-selective PROTAC ZXH-3-26 by adjusting the length and modification site of linker to generate a favorable BRD4-CRBN binding conformation [93]. Besides, a number of other BRD4-based PROTACs have also been developed for cancer therapy [89, 92, 94–98, 100, 137–140].

#### CDK4/CDK6

Cyclin-dependent kinases (CDKs) control cell cycle progression in response to extracellular pro-proliferative signals. Among them, CDK4/6 phosphorylate retinoblastoma protein (Rb) and activate the transcription factor E2F to promote gene transcription, mediating the G1 to S phase transition [141]. In cancer cells, CDK4/6 are usually overactivated by their upstream oncogenes (e.g. c-myc) and serve as potential targets for cancer therapies [142, 143].

In 2019, Zhao et al. exploited the CRBN ligand and palbociclib (a CDK4/6 inhibitor) to synthesis the PROTAC Pal-pom that degraded CDK4/6 with DC_50_ values of 20–50 nM, thus preventing Rb phosphorylation and inducing cell cycle arrest in triple negative breast cancer (TNBC) cells [104]. Subsequently, Jiang et al. obtained the new PROTAC BSJ-02-162 based on Pal-pom by introducing a shorter alkyl chain and removing the 1,2,3-triazole moiety, which degraded both CDK4/6 and IKZF1/3 to exhibit increased anti-proliferative function in mantle cell lymphoma cells [77]. Another CDK inhibitor ribociclib was used into the first orally bioavailable prodrug of PROTAC, which degraded CDK 2/4/6 *in vivo* [105].

The high sequence similarity of CDK4 and CDK6 near their active sites makes them difficult to be distinguished by current CDK inhibitors. Interestingly, increasing evidence demonstrates that PROTAC exhibits good substrate selectivity after its optimization or molecule modifications. For example, Gray’s group optimized linkers to successfully develop the CDK6-selective degrader BSJ-03-123 based on palbociclib [102] and the CDK4-selective degrader BSJ-04-132 based on ribociclib [77]. This selectivity might be caused by the cooperative CDK-CRBN interactions as described in the “neocontacts” of BRD4-based PROTACs [33, 93]. Additionally, through adding oxygen or nitrogen atom to the linker of BSJ-02-162, PROTACs CP-10 and YX-2-107 can selectively degrade CDK6 [78, 144]. Notably, CDK6 exerts its functions in both kinase-dependent and -independent manners, and only its kinase-independent function is required for the growth of Philadelphia-positive acute lymphoblastic leukemia (Ph^+^-ALL). PROTAC YX-2-107 was demonstrated to inhibit CDK6’s kinase-independent function, thus more efficiently suppressing Ph^+^-ALL cells compared to palbociclib (inactive to CDK6’s kinase-independent function) [78]. Except for CRBN, Steinebach et al. found that VHL also had the potential to selectively degrade CDK6 in leukemia, myeloma and breast cancer cells [107].

#### AURORA-a

Aurora kinase A (AURORA-A) drives centrosome separation to induce cell cycle progression from G2 to M phase. Overexpressed AURORA-A could transform normal epithelial cells to cancer cells in mouse models, highlighting AURORA-A as a prior cancer target [145]. The potent AURORA-A inhibitor alisertib is in multiple clinical trials. Besides the catalytic activity, AURORA-A has additional non-catalytic functions that are difficult to target by conventional small molecules, which may explain why some trials exhibit low therapeutic efficacy [146, 147]. To overcome this problem, Adhikari et al. developed a potent AURORA-A degrader JB170 by connecting alisertib to VHL ligand, which induced rapid, durable and highly-specific degradation of AURORA-A in leukemia and neuroblastoma cells [81]. Moreover, AURORA-A degradation by JB170 arrested S-phase progression and this effect was not observed upon kinase inhibition, further supporting the important non-catalytic function of AURORA-A during DNA replication [81].

#### EGFR

Epidermal growth factor receptor (EGFR) is a receptor tyrosine kinase (RTK) that activates several oncogenic signals, promoting cell proliferation and differentiation. Overactivation or gain-of-function mutation of EGFR are prevalent in a variety of epithelial cancers (e.g. breast and lung cancers) [75, 148]. EGFR inhibitors, such as gefitinib, lapatinib and afatinib, have been approved to treat cancers, but severe drug resistance of EGFR inhibitors leads to low clinical response, which may be caused by drug-induced EGFR mutations (e.g. EGFR^L858R^, EGFR^T790M^ or EGFR^C797S^) [149].

Based on lapatinib, gefitinib and afatinib, PROTACs compound 1/3/4 were respectively developed by linking VHL ligands, exhibiting anti-proliferative activity against breast cancer and lung cancer cells [79]. These PROTACs were selective for different EGFRs: compound 1 degraded wild-type or exon-20 insertion EGFRs; compound 2 preferred exon-19 deletion or L858R EGFRs; compound 3 degraded L858R/T790M dual mutant EGFR [79]. Novel EGFR^L858R/T790M^ selective inhibitors XTF-262 and EGFR^T790M/C797S^ selective inhibitors EAI001 were also utilized to synthesis PROTAC 14o and DDC-01-163, respectively, which exhibited anti-proliferative activities in lung cancer cells with corresponding EGFR-mutations [80, 99]. Overall, the selectivity of EGFR inhibitor-based PROTACs was consistent with that of their parental inhibitors [79, 80, 99, 101, 103, 150], so it’s necessary to conduct molecular typing of EGFR before PROTAC treatment.

#### BRAF

The RAF family kinases are key regulators of RAS-RAF-MEK-ERK pathway, transmitting oncogenic signals to promote cell proliferation [75]. Gain-of-function mutations in RAF (e.g. BRAF^V600E^) act as potent drivers of human cancers [151]. BRAF^V600E^ inhibitors have shown great efficacy in cancer therapy, but long-term effectiveness is limited by RTKs and/or RAS activation or by secondary BRAF mutations [152, 153]. PROTAC provides an alternative strategy to therapeutically constrain oncogenic BRAF [60, 154]. Posterna et al. conjugated BRAF inhibitor BI-882370 and CRBN ligand to synthesize the PROTAC P4B, which specifically suppressed melanoma and colorectal cancer cells harboring BRAF^V600E^ or other BRAF mutations [60].

Except for these targets, the following proteins related to cancer cell proliferation could also be targeted by PROATCs: AR [7, 106, 108–111, 155, 156], ALK [22, 52, 113, 116, 157], BLK [118], BRD7/9 [82, 83, 120, 158], CDK2/5 [84], CDK8 [112], CDK9 [38, 114, 115, 117, 159], Cdc20 [119], c-Met [79], CREPT [121], CYP1B1 [122], DHODH [123], ER [89, 124, 126, 128, 160], ERK1/2 [26], FLT-3 [132, 161], HER2 [79], MEK1/2 [85, 86, 162], KRAS^G12C^ [87, 163], GSPT1 [125], PLK1 [127], SLC9A1 [129], TACC3 [130], TRIM24 [131], TRKA/C [133, 164], Wee1 [88], α_1A_-AR [134].

### Targeting cancer apoptosis

**Fig. 4:**
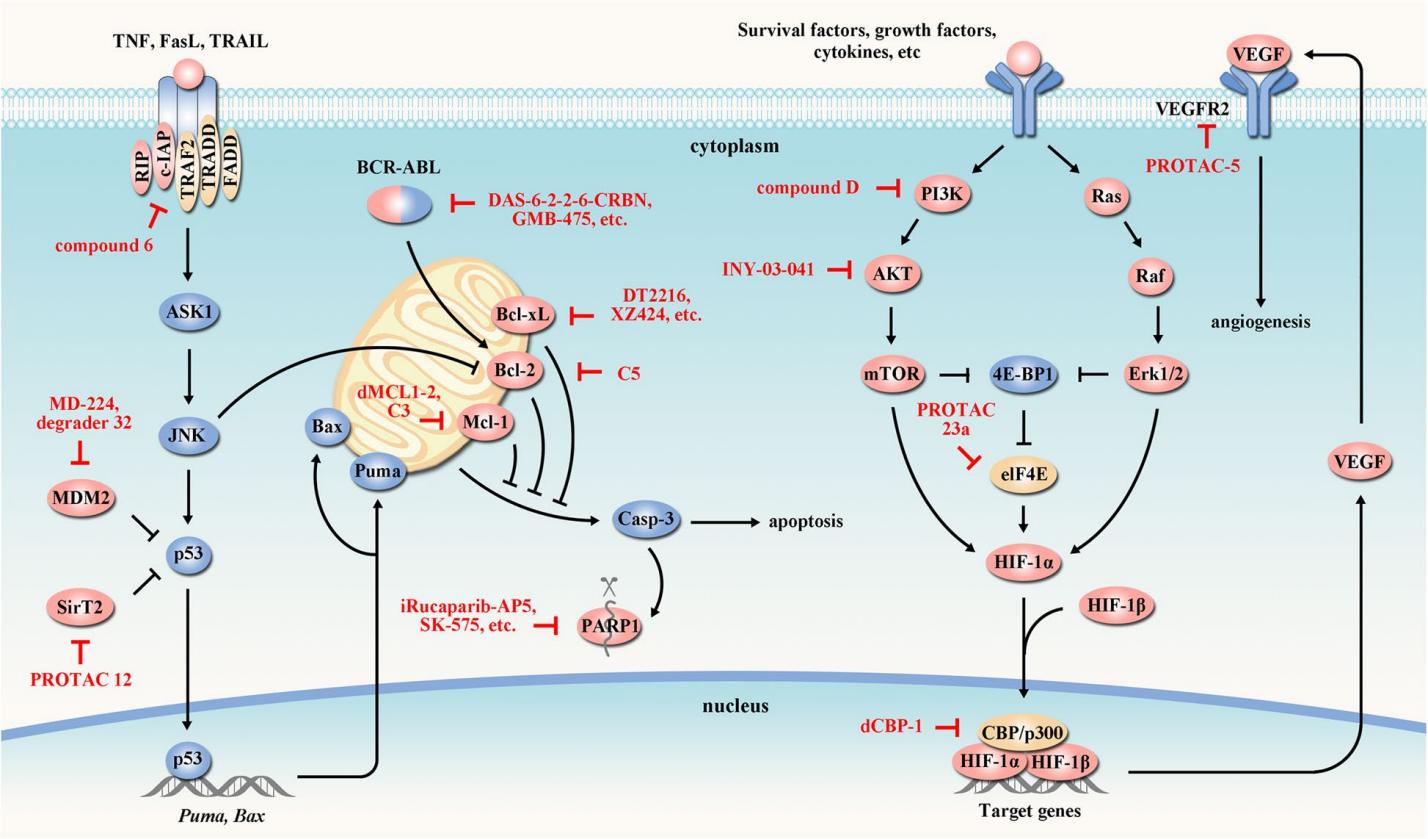
PROTACs targeting apoptosis and angiogenesis. Apoptosis is accomplished by downregulation of antiapoptotic proteins (e.g. Bcl-2, Bcl-6, Bcl-xL, Mcl-1, etc) or upregulation of proapoptotic factors (e.g. Puma, Bax). This process can be regulated by various proteins (e.g. p53) and signaling pathways (e.g. PI3K/AKT). PI3K/AKT activation also promotes VEGF expression and angiogenesis. These key elements involved in cancer apoptosis and angiogenesis can be targeted by PROTACs (red arrow). Tumor-suppressor proteins are indicated in blue and oncogenic proteins are indicated in red. In the presented pathways, PROTACs have been developed targeting Bcl-xL [167, 168], PARP1 [169, 170], BCR-ABL [171, 172], AKT [173], Bcl-2 [174], c-IAP [175], eIF4E [176], Mcl-1 [174, 177], MDM2 [178, 179], PI3K [180], CBP/p300 [181], SirT2 [182] and VEGFR2 [183]

Apoptosis (or programmed cell death) is an evolutionarily conserved process that maintains tissue homeostasis upon the simulation by cellular stress, DNA damage and immune surveillance. However, cancer cells upregulate anti-apoptotic proteins (e.g., Bcl-2 and Bcl-xL) or downregulate pro-apoptotic factors (e.g., Puma, Bax) to evade apoptosis, supporting their abnormal survival, therapeutic resistance and cancer recurrence [165, 166]. Therefore, targeting apoptosis could initiate programmed cell death of cancer cells and improve their response to anticancer drugs (Fig. 4) (Table 3).

**Table 3:** The structures of PROTAC molecules targeting apoptosis or angiogenesis in cancers. (red: POI ligand; yellow: E3 ligand)

Target	PROTAC	PROTAC structure	Cancer	Ref	Target	PROTAC	PROTAC structure	Cancer	Ref
Bcl-xL	DT2216		T-ALL	[167]	BCR-ABL	DAS-6-2-2-6-CRBN		CML	[171]
Bcl-xL	XZ739		T-ALL	[184]	BCR-ABL	Azo-PROTAC-4C		CML	[19]
Bcl-xL	compound 8a		TL	[185]	BCR-ABL	SNIPER(ABL)-38		CML	[89]
Bcl-xL	XZ424		T-ALL	[168]	BCR-ABL	compound 19		CML	[186]
Bcl-xL	PROTAC 6		AML	[187]	BCR-ABL	GMB-475		CML	[172]
PARP1	compound 2		CRC	[188]	BCR-ABL	GMB-805		CML	[189]
PARP1	iRucaparib-AP5		CC, RCC, BC, PC	[170]	BCR-ABL	SIAIS178		CML	[190]
PARP1	compound 3		TNBC	[191]	BCR-ABL	BT1		CML	[192]
PARP1	SK-575		BC, CRC, PC, PaC	[169]	AKT	INY-03-041		BC	[173]
Bcl-2	C5		CC, CML, NSCLC	[174]	eIF4E	PROTAC 23a		TNBC, CML	[176]
Bcl-6	PROTAC 15		DLBCL	[193]	HDAC1/2/3	PROTAC 4		CRC	[194]
c-IAP	compound 6		FS	[175]	HDAC1/2/3	XZ9002		TNBC	[195]
CK2	PROTAC 2		TNBC, NSCLC	[196]	HDAC6	PROTAC 4		OSCC, GB	[197]
CRABP I/II	compound 4b		NB	[8]	HDAC6	degrader 12d		MM	[198]
CRABP I/II	compound 6		FS	[175]	HDAC6	NP8		MM, CC	[199]
CRABP I/II	β-NF-ATRA		BC, NB	[140]	HDAC6	NH2		MM, CC	[200]
eEF2K	compound 11l		BC	[201]	HDAC6	P1		MM	[202]
HDAC6	compound 3j		MM	[203]	CBP/ p300	dCBP-1		MM	[181]
Mcl-1	dMCL1-2		MM	[177]	RIPK2	PROTAC_RIPK2		BC, AML	[17]
Mcl-1	C3		CC, CML, NSCLC	[174]	SGK3	SGK3-PROTAC1		BC	[204]
MDM2	MD-224		ALL, AML	[179]	SirT2	PROTAC 12		CC	[182]
MDM2	degrader 32		ALL	[178]	VEGFR2	PROTAC-5		/	[183]
PI3K	compound D		HCC	[180]					

#### Bcl-xL

Bcl-xL inactivates the intrinsic apoptotic pathway to promote cell survival. Overexpression of Bcl-xL occurs in many tumor cells and is highly correlated with the resistance to cancer therapy, so Bcl-xL is a well-validated cancer target [165]. However, the low target engagement and dose-limiting thrombocytopenia limits the use of Bcl-xL inhibitors (e.g. ABT263 and A-1155463) as safe and effective anticancer agents [205].

Zhou and his coworkers linked ABT263 to VHL ligand to develop the PROTAC DT2216, which effectively degraded Bcl-xL and suppressed Bcl-xL-dependent leukemia cells *in vitro* and *in vivo*, without causing thrombocytopenia due to the poor expression of VHL in platelets [167]. Since CRBN is poorly expressed in platelets, they designed another PROTAC XZ739 containing CRBN ligand and ABT263, treating T cell acute lymphoblastic leukemia (T-ALL) with less toxicity to platelets [184]. Because VHL and CRBN expressions are extremely low in cutaneous T-cell lymphoma (CTCL) cells, the activity of VHL- and CRBN-based Bcl-xL PROTACs against CTCL were unfavorable. Zhou group further designed PROTAC 8a involving the ligand of IAP E3 ligase (with high level in CTCL) to efficiently degrade Bcl-xL in CTCL cells [185]. The selective Bcl-xL inhibitor A-1155463 was also utilized to develop XZ424 and PROTAC 6, showing increased selectivity in Bcl-xL-dependent T-ALL cells [168, 187].

#### PARP1

Poly(ADP-ribose) polymerase 1 (PARP1) participates in DNA damage repair to maintain genomic stability, and is overexpressed in human cancers to evade apoptosis [166]. Small-molecule PARP1 inhibitors, such as niraparib, rucaparib and olaparib, have been developed to treat cancers [206]. However, these inhibitors prevent PARP1 from dissociating DNA lesions to block DNA replication, leading to high cytotoxicity to normal cells [206].

In 2019, by connecting niraparib and the MDM2 ligand nutlin-3, Zhao et al. synthesized the PARP1-targeting PROTAC compound 3 to induce significant apoptosis of TNBC cells without cytotoxicity against normal cells [191]. Olaparib was also used to design CRBN-recruiting PROTACs to trigger apoptosis in multiple cancers [169, 188]. To improve selectivity, Wang et al. utilized rucaparib (a selective PARP1 inhibitor) and CRBN ligand to develop PARP1 degrader iRucaparib-AP5, which exerted highly specific PARP1 degradation in cervical, breast, renal and prostate cancer cells [170].

#### BCR-ABL

The oncogenic fusion kinase BCR-ABL activates the anti-apoptotic protein Bcl-2 to protect mitochondria from DNA-damaged signals and prevent apoptosis in chronic myelogenous leukemia (CML) [207, 208]. BCR-ABL inhibitors (e.g. dasatinib, ponatinib and imatinib) have successfully treated CML patients. But lifelong drug administration is required due to the persistent CML stem cells that rely on BCR-ABL’s kinase-independent function for survival [171, 209]. Moreover, BCR-ABL mutations can also cause drug resistance [210].

Lai et al. synthesized the BCR-ABL degrader DAS-6-2-2-6-CRBN containing dasatinib and CRBN ligand, which showed potent degradation of BCR-ABL and growth inhibition in CML cells [171]. Other E3 ligases including IAP, VHL and RNF114 were also recruited by dasatinib-based PROTACs that achieved effective BCR-ABL degradation to suppress CML cells [89, 190, 192]. Ponatinib and two novel BCR-ABL inhibitors (GNF5 and ABL001) were utilized into PROTACs development, and the obtained degraders showed increased degradation ability and better selectivity with less adverse effects [172, 186, 189].

### Targeting cancer angiogenesis

Tumors require neovasculature, generated by angiogenesis, to supply nutrients and oxygen as well as to evacuate metabolic wastes and carbon dioxide [73]. Angiogenesis is triggered by hypoxia that activates the expression of multiple growth factors, such as vascular endothelial growth factor (VEGF), a pivotal growth factor that specifically recognizes vascular endothelial growth factor receptor (VEGFR) to induce the formation of neovasculature (Fig. 4) [211]. The blockade of VEGF/VEGFR signaling to suppress angiogenesis has been developed for cancer therapy (Fig. 4, Table 3).

VEGFR-2 is the main VEGFRs to mediate proliferation and angiogenesis of vascular endothelial cells, and targeting VEGFR2 is a promising strategy for cancer treatment. Based on the VEGFR-2 inhibitor S7, Shan et al. developed PROTAC-2 and PROTAC-5 to exhibit potent VEGFR-2 elimination and anti-proliferative activity in human umbilical vein endothelial cells. Moreover, these PROTACs had low cytotoxicity to HEK-293 cells (human embryonic kidney cells, VEGFR-2 negative), displaying excellent safety to VEGFR-2 negative cells [183].

### Targeting cancer immunity and inflammation

**Fig. 5:**
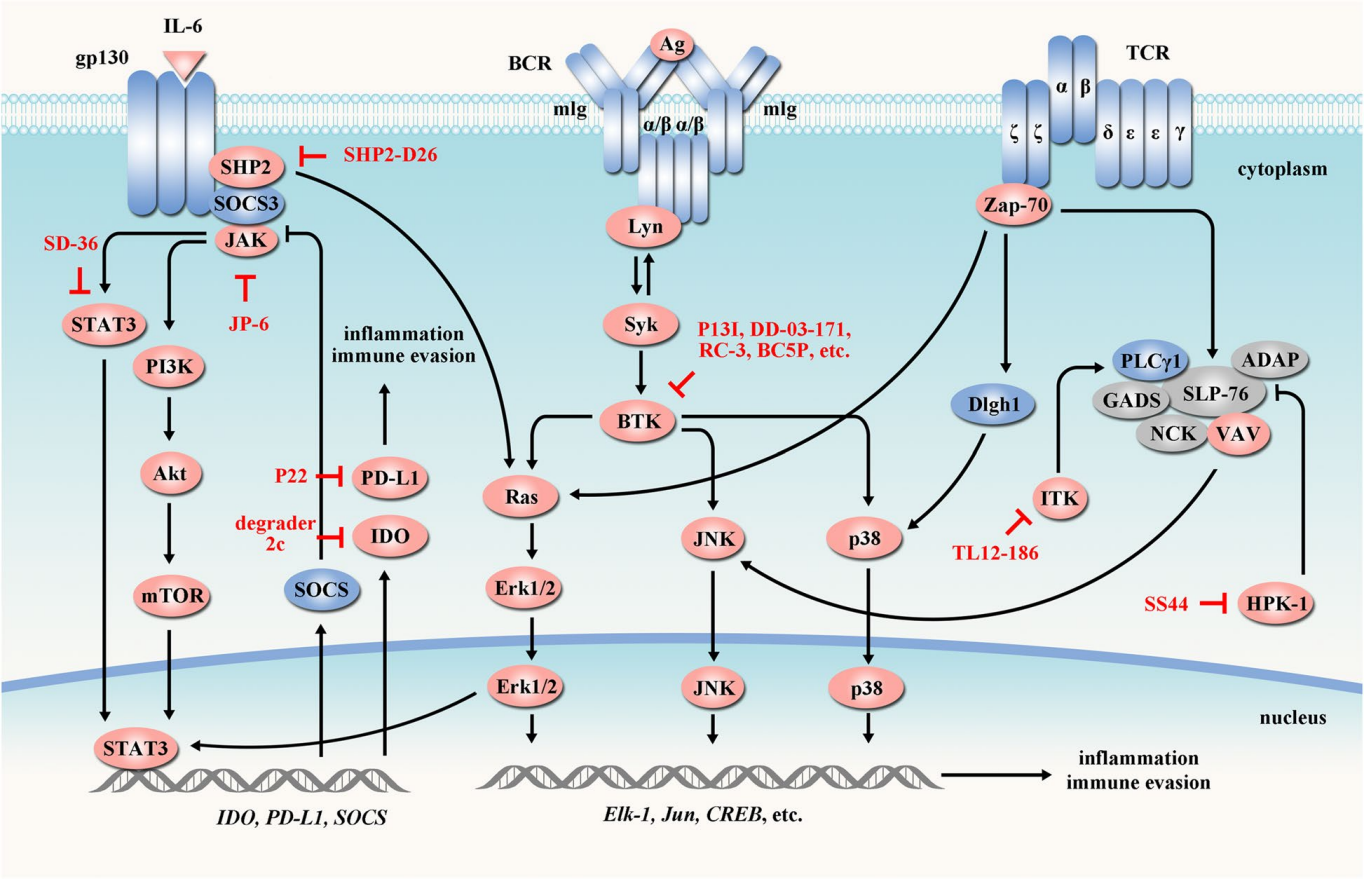
PROTACs targeting cancer immune evasion and inflammation. Cancer cells promote immune evasion and inflammation by reprogramming tumor microenvironment that involves BCR, TCR and JAK-STAT pathways. These components in cancer immune evasion and inflammation can be targeted by PROTACs (red arrow). Tumor-suppressor proteins are indicated in blue and oncogenic proteins are indicated in red. In the presented pathways, PROTACs have been developed targeting PD-L1 [215], BTK [216–219], STAT3 [57], HPK1 [220], IDO1 [221], ITK [161], JAK [222] and SHP2 [223]

To sustain cell survival, cancer cells induce inflammation and immune evasion by reprogramming tumor microenvironment that involves regulatory cells (e.g., regulatory T cells), B-cell receptor (BCR) signaling and T-cell receptor (TCR) signaling [212–214] (Fig. 5).

Immunotherapies by the immune-checkpoint inhibitors are new therapeutics that relieve immunosuppression and enable immune-mediated tumor clearance [224]. However, some patients have innate or acquired resistance to immunotherapies. To overcome these problems, PROTACs targeting immunity and inflammation have been developed (Table 4).

**Table 4:** The structures of PROTAC molecules targeting cancer immune evasion or inflammation. (red: POI ligand; yellow: E3 ligand)

Target	PROTAC	PROTAC structure	Cancer	Ref	Target	PROTAC	PROTAC structure	Cancer	Ref
PD-L1	P22		NSCLC TNBC, M	[215]	FKBP12	dFKBP-1		AML	[12]
BTK	DD-04-015		DLBCL	[161]	FKBP12	dTAG-13		AML	[225]
BTK	P13I		BL, DLBCL, MCL	[216]	FKBP12	RC32		T-ALL, BL, PC, BC, CC	[226]
BTK	RC-3		BL, MCL, CML	[217]	FKBP12	KB02-SLF		/	[97]
BTK	RC-1		AML, MCL	[227]	HPK1	SS44		BL, MM, CML, et al	[220]
BTK	BC5P		AML	[219]	IDO1	degrader 2c		CC	[221]
BTK	DD-03-171		MCL, DLBCL	[218]	IKZF1/3	DD-03-171		MCL, DLBCL	[218]
BTK	compound 10		BL, AML	[228]	IRAK4	degrader-5		DLBCL	[229]
BTK	PROTAC 7		BL, CML	[118]	ITK	TL12-186		AML, T-ALL	[161]
STAT3	SD-36		AML, ALCL	[57]	JAK	JP-6		AML	[222]
CD147	compound 6a		M	[230]	Lin28	ORN3P1		CML	[18]
PDE4	SNIPER(PDE4)-9		FS	[89]	PRC2	PROTAC 1		DLCBL	[231]
PDEδ	compound 17f		CRC	[232]	PRMT5	MS4322		BC	[233]
PDEδ	PROTAC 3		T-ALL, PaC, CC	[234]	Rpn13	WL-40		MM	[235]
Pirin	CCT367766		OC	[236]	SHP2	SHP2-D26		EC, AML	[223]
PRC2	UNC6852		CC, DLCBL	[237]	TBK1	PROTAC 3i		NSCLC	[238]

#### PD-L1

Programmed death-ligand 1 (PD-L1) is frequently overexpressed in cancer cells. The binding of PD-L1 on cancer cells to its receptor programmed death 1 (PD-1) on T cells counteracts T cell-activating signals, inhibiting anti-tumor immunity and promoting immune escape [239]. Chen group used BMS-1198 (a small-molecule PD-L1 inhibitor) and pomalidomide (a CRBN ligand) to synthesize PROTAC P22, which moderately degraded PD-L1 in lung and breast cancer cells [215]. Thus, it’s possible to develop PD-L1-targeting PROTAC based on small-molecule PD-L1 inhibitors. However, due to the hydrophobic and flat binding pocket of PD-L1, there are few known small-molecule PD-L1 inhibitors, so it’s challenging to develop effective PD-L1 PROTAC currently.

#### BTK

Bruton’s tyrosine kinase (BTK) is a non-receptor tyrosine kinase, playing pivotal roles in B-cell development and immune responses. BTK inhibitors (e.g. ibrutinib) have been developed to treat chronic lymphocytic leukemia (CLL) and mantle cell lymphoma (MCL) by blocking BCR signaling and regulating innate/adaptive immunity [240]. But many patients exhibit drug resistance due to BTK mutations in the ibrutinib binding site (BTK^C481S^).

Based on ibrutinib and CRBN ligand, Rao group developed PROTACs P13I and L18I, two irreversible covalent PROTACs, to degrade wide-type and C481S-mutant BTKs and suppress diffuse large B cell lymphoma (DLBCL) and MCL cells [216, 241]. As P13I and L18I formed irreversible covalent bonds with BTK, so they did not follow “event-driven mechanism”, even though they used the PROTAC-like structure. To solve this problem, another two groups designed PROTACs RC-1 and RC-3 using cyano-acrylamide moiety to shape up reversible covalent bonds with BTK, exhibiting enhanced selectivity and efficacy over irreversible PROTACs [217, 227]. Additionally, a new generation of non-covalent BTK inhibitors (e.g., RN486 and CGI1746) was utilized to develop PROTACs (e.g., DD-04-015 and DD-03-171) that efficiently degraded BTKs and inhibited cancer cell growth [161, 218]. Intriguingly, Calabrese group found that alleviation of steric clashes between BTK and CRBN by adjusting PROTAC linker length allowed potent BTK degradation in the absence of thermodynamic cooperativity [228], indicating increased BTK-PROTAC-IAP ternary complex stability was not always related to increased degradation efficiency [219]. However, its underlying mechanism remains obscure.

### Targeting cancer metastasis

**Fig. 6:**
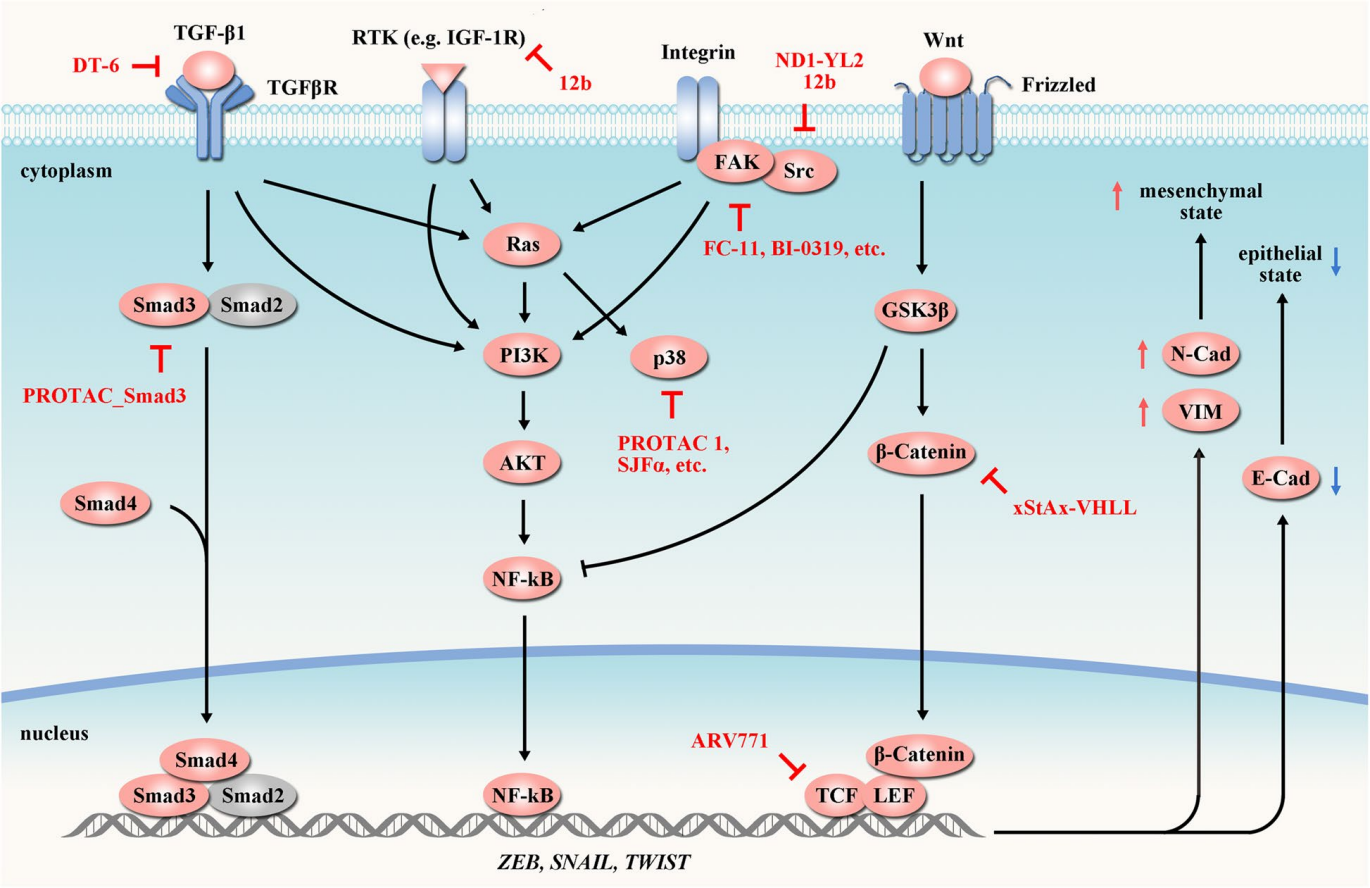
PROTACs targeting cancer metastasis. Activation of Integrin/FAK/PI3K/AKT, TGF-β/SMAD and Wnt/β-catenin pathways significantly increase the expression of pro-EMT transcription factors (e.g. ZEB, SNAIL and TWIST), leading to the downregulation of E-cadherin (E-Cad) that maintains epithelial integrity, and the upregulation of N-cadherin (N-Cad) and vimentin (VIM) that implicate in motility and invasion. These key elements involved in metastasis can be targeted by PROTACs (red arrow). Tumor-suppressor proteins are indicated in blue and oncogenic proteins are indicated in red. In the presented pathways, PROTACs have been developed targeting FAK [246, 247], IGF-1R [248], p38 [249, 250], Smad3 [251], Src [248, 252], TCF [253], TGF-β1 [254] and β-catenin [255]

Tumor cells extravasate, disseminate and successfully colonize distant organs from the primary foci via circulatory systems to achieve metastasis, causing ~ 90% of cancer deaths worldwide [242, 243]. Epithelial-to-mesenchymal transition (EMT) is a key step during metastasis and can be activated by several upstream cellular signaling pathways including Integrin/FAK/PI3K/AKT axis (Fig. 6) [243–245]. In the past decades, PROTACs targeting EMT-related proteins have been developed to manage cancer metastasis (Table 5).

**Table 5:** The structures of PROTAC molecules targeting cancer metastasis. (red: POI ligand; yellow: E3 ligand)

Target	PROTAC	PROTAC structure	Cancer	Ref	Target	PROTAC	PROTAC structure	Cancer	Ref
FAK (PTK2)	PROTAC-3		TNBC, PC	[256]	p38	NR-7h		BC, CRC, CC	[257]
FAK (PTK2)	BI-0319		HCC	[246]	Smad3	PROTAC_Smad3		RCC	[251]
FAK (PTK2)	BI-3663		HCC	[246]	Src	ND1-YL2		TNBC	[252]
FAK (PTK2)	FC-11		OC, BC, PC, BL	[247]	Src	12b		BC, NSCLC	[248]
IGF-1R	12b		BC, NSCLC	[248]	TCF	ARV771		DLBCL	[253]
p38	SJFα		BC, CC	[249]	TGF-β1	DT-6		HCC, BC, NSCLC	[254]
p38	PROTAC 1		BC, CC	[250]	β-catenin	xStAx-VHLL		CRC	[255]

#### FAK

Focal adhesion kinase (FAK) is one of the most prominent effectors of integrin signaling. Overexpressed FAK, correlating with poor clinical outcome, drives cancer invasion and migration through exerting both kinase-dependent and independent functions [258–260]. Several FAK kinase inhibitors have been developed, such as defactinib, BI-4464 and PF-562271 [261]. Nevertheless, the critical kinase-independent scaffolding function of FAK is beyond the ability of current inhibitors [262].

Cromm et al. used the clinical candidate defactinib as the FAK ligand and (S,R,S)-AHPC as the VHL ligand to prepare a selective FAK degrader PROTAC-3. PROTAC-3 dramatically suppressed FAK signaling as well as FAK-mediated cell migration and invasion in TNBC and prostate cancer cells [256]. Based on small-molecule FAK inhibitor BI-4464 and CRBN ligand pomalidomide, Popow et al. presented a highly selective PROTAC BI-3663 to hijack UPS for FAK degradation, showing a DC_50_ of 30 nM in a panel of hepatocellular carcinoma cell lines [246]. In addition, FAK inhibitor PF562271 was also included in PROTAC study, leading to the establishment of PROTAC FC-11 that exhibited rapid FAK degradation with picomolar DC_50_ in several cancer cells [247].

## Guidelines for PROTAC design

Developing anticancer PROTAC aims to improve the effectiveness and precision of targeted cancer therapy. In the molecular design of PROTACs, many critical issues about POI ligand, E3 ligand and linker should be comprehensively considered, such as specificity, solubility, stability, drug safety and bioavailability.

For POI/E3 ligands, the known ligands as well as the newly designed ligands based on 3D structure of POI/E3 could be used in PROTAC design. Notably, the ligand with high target affinity is not favorable, because this makes the ligand difficult to dissociate from target protein and is more likely to exert “occupancy-driven mechanism” instead of “event-driven mechanism” [250, 263]. A transient or low-abundance “POI-PROTAC-E3” ternary complex is enough to achieve adequate degradation, thus the ligand with low target affinity without affecting the assembly of ternary complex is acceptable. Moreover, selective inhibitors could be utilized to increase the precision of PROTACs, while the multitargeted inhibitors are also useful to develop PROTAC degraders that exert anticancer activities by simultaneously degrading multiple proteins [161]. Besides, there are ~ 600 E3 ligases in human and their expressions usually exhibit tissue−/tumor-specific, so selecting appropriate E3 ligase/E3 ligand system should consider the cellular context of tumors to increase the efficacy and reduce the toxicity [27, 36].

For the linker, the first issue is to define the ligand site that binds to the linker. The protein- or cell-based biological assays should be performed to test the activity of ligands with chemical modifications at different sites, aiming to find the promising sites that maintain the ligand’s function. Secondly, since the physical and chemical properties of linkers affect PROTAC’s selectivity and efficiency via adjusting the POI-E3 interface, a series of linkers with different lengths and chemical compositions should be designed, simulated (e.g., by structural modelling or molecular simulation), synthesized and biologically evaluated. Importantly, for the convenience in preparation and purification of PROTACs, the hydrophilicity/liposolubility of linker needs to match the properties of POI ligand and E3 ligand.

In addition, since PROTACs with high M.W. may influence their bioavailability, the idea of CLIPTACs that design a pair of smaller precursors is feasible to increase cell permeability [26]. It’s also recommended to use computer-aided drug design (CADD) software (e.g. Discovery Studio or Schrodinger Suites) to *in silico* predict the solubility and ADMET (Absorption, Distribution, Metabolism, Excretion and Toxicity) properties of molecules before PROTAC design.

## Conclusions and prospects

From the establishment of the PROTAC concept in 2001, extensive efforts have been devoted to improving the efficacy, expanding the target scope and overcoming the disadvantages of PROTAC. Therefore, PROTAC has become an attractive technique for cancer treatment. Until now, many PROTACs have been developed to control cancer progression, exhibiting clinical potential in cancer therapy. However, there is still a demand to accelerate the development of PROTACs.

Expanding the POI spectrum is urgent for cancer therapy. Currently, although inhibitors of some proteins (e.g. kinases) have been successfully developed, there are many oncogenic proteins (e.g. RBPs and DBPs) that can’t be targeted by small molecules. Interestingly, taking advantage of the fact that RBPs and DBPs bind to specific nucleotide sequences, researchers utilized oligonucleotides as POI ligands to develop the RNA-PROTAC and TF-PROTAC that induced the degradation of RBPs and DBPs [18, 55]. Therefore, these techniques open up a new direction for targeting undruggable pathogenic proteins. In future, the design and optimization of these oligonucleotide-based PROTACs, targeting oncogenic RBPs (e.g. IGF2BPs and YBX1) and DBPs (e.g. c-myc and STAT3), should be extensively investigated for targeted cancer therapy.

Less than 10 of ~ 600 E3 ligases have been utilized in PROTAC so far, other E3 ligases could be considered to develop new PROTACs. For example, Cotton et al. established antibody-based PROTACs (AbTACs) that the recombinant bispecific antibodies recruit the membrane-bound E3 ligase RNF43 for the degradation of the cell-surface protein PD-L1 [264]. Moreover, the proteasome-independent protein degradation systems (including endosome, lysosome or autophagosome systems) have been harnessed to develop novel targeted degradation techniques, such as lysosome-targeting chimera (LYTAC), autophagy-targeting chimera (AUTAC) and autophagosome-tethering compound (ATTEC) [265–267], providing different strategies for targeted cancer therapy. Additionally, the ribonuclease targeting chimera (RIBOTAC) used RNA-targeting small molecules and RNase L to accomplish the degradation of intracellular RNAs [268, 269], suggesting a new idea for the degradation of oncogenic RNAs for cancer therapy. Therefore, PROTAC and related degradation techniques are powerful tools for specifically degrading oncogenic proteins or RNA molecules and will be used clinically for cancer therapy.

## Data Availability

Not applicable.

## Abbreviations

ALCL: Anaplastic large cell lymphoma
ALL: Acute lymphoblastic leukemia
AML: Acute myeloid leukemia
BC: Breast cancer
BL: Burkitt’s lymphoma
BRD4: Bromodomain-containing protein 4
BTK: Bruton’s tyrosine kinase
CC: Cervical cancer
CDK: Cyclin-dependent kinase
CML: Chronic myelogenous leukemia
CRBN: Cereblon
CRC: Colorectal cancer
DLBCL: Diffuse large B cell lymphoma
EC: Esophageal cancer
EGFR: Epidermal growth factor receptor
FAK: Focal adhesion kinase
FS: Fibrosarcoma
GB: Glioblastoma
GC: Gastric cancer
HCC: Hepatocellular cancer
IAP: Inhibitor-of-apoptosis-protein
LC: Lung cancer
M: Melanoma
MCL: Mantle cell lymphoma
MM: Multiple myeloma
MRT: Malignant rhabdoid tumor
NB: Neuroblastoma
NSCLC: Non-small cell lung cancer
OC: Ovarian cancer
OS: Osteosarcoma
OSCC: Oral squamous cell carcinoma
PaC: Pancreatic cancer
PARP1: Poly(ADP-ribose) polymerase 1
PC: Prostate cancer
PD-L1: Programmed death-ligand 1
POI: Protein of interest
PROTAC: Proteolysis-targeting chimeras
RCC: Renal cell cancer
SS: Synovial sarcoma
T-ALL: T cell acute lymphoblastic leukemia
TL: T-cell lymphoma
TNBC: Triple negative breast cancer
Ub: Ubiquitin
UPS: Ubiquitin-proteasome system
VEGFR: Vascular endothelial growth factor receptor
VHL: Von Hippel-Lindau
